# Intracanal Calcification Following Apexification: A Case Series

**DOI:** 10.7759/cureus.109588

**Published:** 2026-05-25

**Authors:** Neha Chauhan, Mridula Goswami, Archana Singh, Rimshheanam Rimshheanam, Akansha Gupta

**Affiliations:** 1 Pediatric and Preventive Dentistry, Maulana Azad Institute of Dental Sciences, New Delhi, IND

**Keywords:** apexification, calcium hydroxide, endodontics, iodoform, tooth calcification

## Abstract

Apexification is a widely employed procedure for managing immature permanent teeth with necrotic pulps, utilizing calcium hydroxide (Ca(OH)₂) and iodoform combinations for canal disinfection and apical barrier formation. While these medicaments are effective, prolonged placement due to missed follow-up appointments may predispose to intracanal calcification, complicating subsequent canal negotiation and obturation. This case series reports three pediatric patients who presented with pain in mandibular permanent first molars exhibiting immature apices (Cvek’s stage 4) and periapical radiolucencies. All patients had previously undergone apexification with a Ca(OH)₂-iodoform medicament (Metapex, Meta Biomed Co. Ltd., Cheongju, Korea) but missed scheduled follow-up appointments, reporting after six to 10 months. At recall, clinical symptoms had subsided, and radiographs showed healing of the periapical lesions. However, during re-instrumentation, extensive intracanal calcifications and calcific barriers were observed in the apical third, obstructing complete canal negotiation and interfering with working length determination. This case series emphasizes the importance of strict follow-up compliance and cautious monitoring when employing long-term intracanal medicaments in pediatric endodontics.

## Introduction

The fundamental objective of pulp therapy is to preserve the structural and functional integrity of the dentition and its supporting tissues while maintaining pulp vitality in teeth affected by caries, trauma, or any other pathology. This is particularly important in young permanent teeth with incompletely developed roots, where maintaining pulp vitality is essential for continued root maturation [1]. Endodontic treatment in pediatric dentistry is directed toward preserving or restoring pulp vitality, facilitating root development, and ensuring the long-term retention of the tooth within the arch. Depending on the pulpal status and stage of root development, treatment approaches in pediatric endodontics may range from vital pulp therapies, such as indirect pulp capping, direct pulp capping, and pulpotomy, to non-vital procedures, including apexification and regenerative endodontic procedures (REPs) [2].

Regenerative endodontic techniques are biologically based procedures that employ tissue engineering principles, including stem cell recruitment, growth factors, and scaffolds, to reestablish pulp vitality and promote root maturation in immature permanent teeth with necrotic pulps, providing superior outcomes to conventional apexification [3]. Apexification is a procedure that involves thorough canal disinfection, removal of necrotic tissues, and placement of biocompatible agents into the canal of an immature non-vital permanent tooth to induce the formation of a mineralized apical barrier and enable subsequent root canal obturation [4,5].

Medicaments used in pediatric endodontics to achieve disinfection of root canals, relieve symptoms, and promote apical barrier formation include calcium hydroxide (Ca(OH)₂), its combination with iodoform, and more recent advancements such as mineral trioxide aggregate (MTA), Biodentine, etc. [1].

Among these, the calcium hydroxide-iodoform combination is most commonly used because of its therapeutic benefits, which include promoting periapical healing, offering potent antimicrobial action and disinfection of the root canal, and its ability to induce hard tissue properties that make it suitable for use in children [6,7]. The therapeutic mechanism of this combination is largely attributable to calcium hydroxide, which disrupts microbial viability through a highly alkaline pH, neutralizes acidic metabolites, and facilitates hard tissue deposition via calcium ion release. The synergistic action of iodoform extends antimicrobial efficacy, while silicone oil ensures sustained release [5,8].

Despite its widespread success, it has been observed that calcium hydroxide, when used as an intracanal medicament in regenerative endodontic therapies, induces intracanal calcifications along with root canal formation in immature teeth [9,10]. This apical barrier formation should be differentiated from intracanal calcification, which represents unintended calcified tissue deposition within the canal lumen. In apexification procedures performed using Ca(OH)₂, failure of the patient to report for follow-up appointments may result in intracanal calcification, which may complicate canal negotiation and subsequent root canal obturation [9]. This case series presents three instances of immature permanent teeth in which intracanal calcifications developed following the use of a calcium hydroxide-iodoform combination, detailing the clinical management strategies employed and follow-up observations in each case.

## Case presentation

This case series comprises patients who reported to the Department of Pediatric and Preventive Dentistry with complaints of pain in the mandibular molar region. Detailed case histories, clinical examinations, and preoperative radiographic assessments (radiovisiography, RVG) were performed for all cases to evaluate the status of the teeth. The patients had no contributory medical or trauma history. The preoperative radiographs revealed immature root apices (Cvek’s stage 4) and periapical radiolucencies (periapical index scores ranging from 3 to 4). All patients had missed scheduled follow-up visits and reported after intervals of six to 10 months with pain in relation to mandibular permanent first molars (International Caries Detection and Assessment System score of 6). Based on the clinical and radiographic findings, all cases were diagnosed with symptomatic apical periodontitis and planned for apexification. Informed consent from the parents and informed assent from the patients were obtained prior to treatment.

Case 1

**Figure 1:**
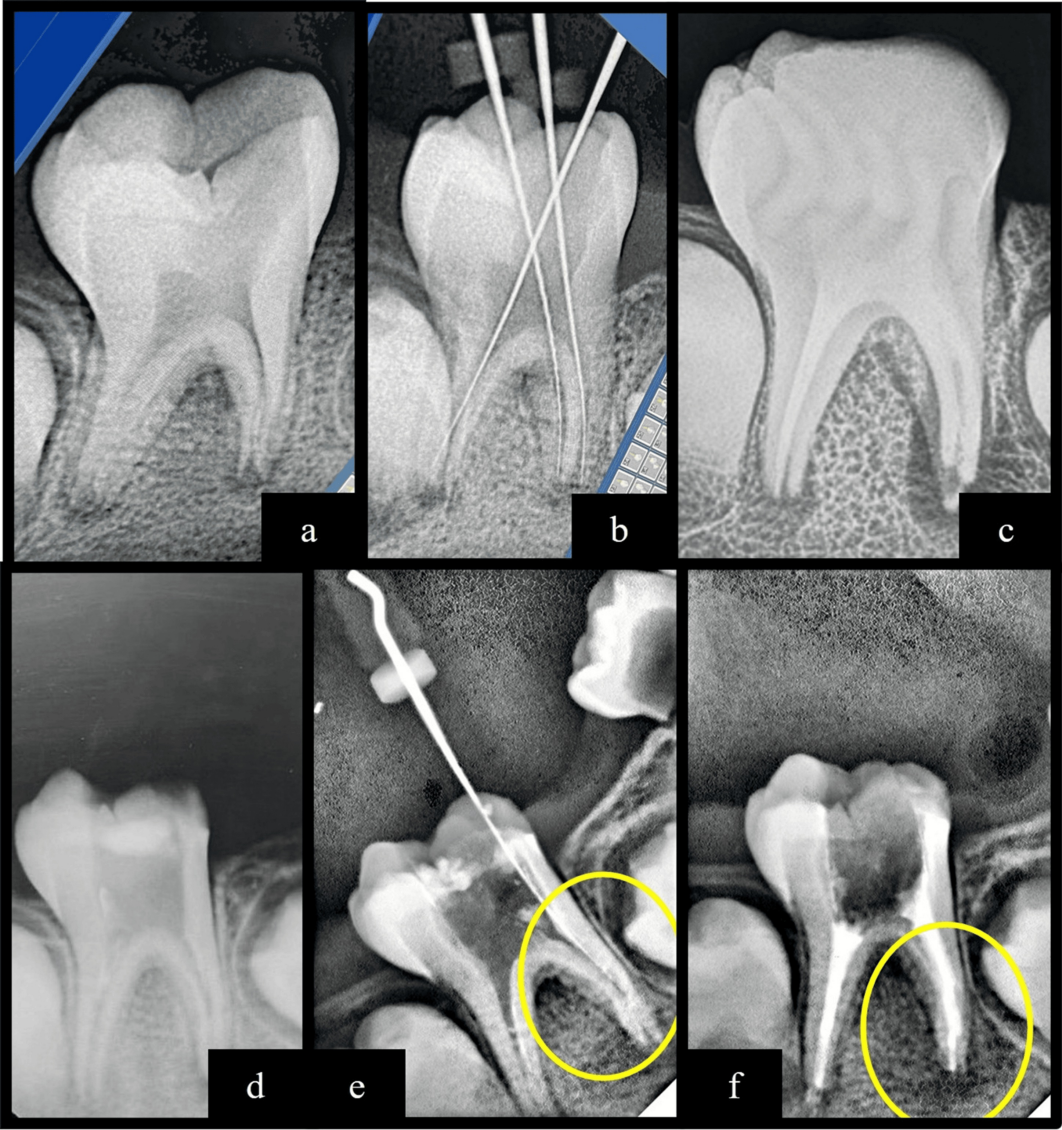
(a) Preoperative radiograph with respect to tooth #46. (b) Working length determination. (c) Metapex medicament for apexification (duration: six months). (d) Metapex removal. (e) Working length determination showing calcification after Metapex removal. (f) Completion of root canal therapy.

An eight-year-old female patient presented with persistent severe pain in the right lower back tooth region for three days. Intraoral examination revealed that tooth #46 was grossly carious and tender on percussion. RVG showed a periapical lesion with ill-defined borders involving the apical aspect of tooth #46, along with open apices. The tooth did not respond to electric and cold pulp testing (Endo-Ice, Coltene/Whaledent Inc., Akron, Ohio, USA) and was planned for apexification. At the first appointment, access opening and cleaning of the canals were performed using normal saline irrigation, followed by placement of Metapex (Meta Biomed Co. Ltd., Cheongju, Korea) as an intracanal medicament and sealing with a temporary restorative material (Cavit-G, 3M ESPE, Seefeld, Germany). The patient failed to attend the scheduled follow-up appointment and returned after six months. At recall, the tooth was asymptomatic, and radiographic evaluation showed healing of the periapical lesion. The case was scheduled for completion of root canal therapy (obturation). During re-instrumentation and canal debridement, hard tissue deposits were observed obliterating the pulp canals in the apical third of the mesiobuccal and mesiolingual canals, hindering further canal negotiation up to the apex (Figure 1).

Case 2

**Figure 2:**
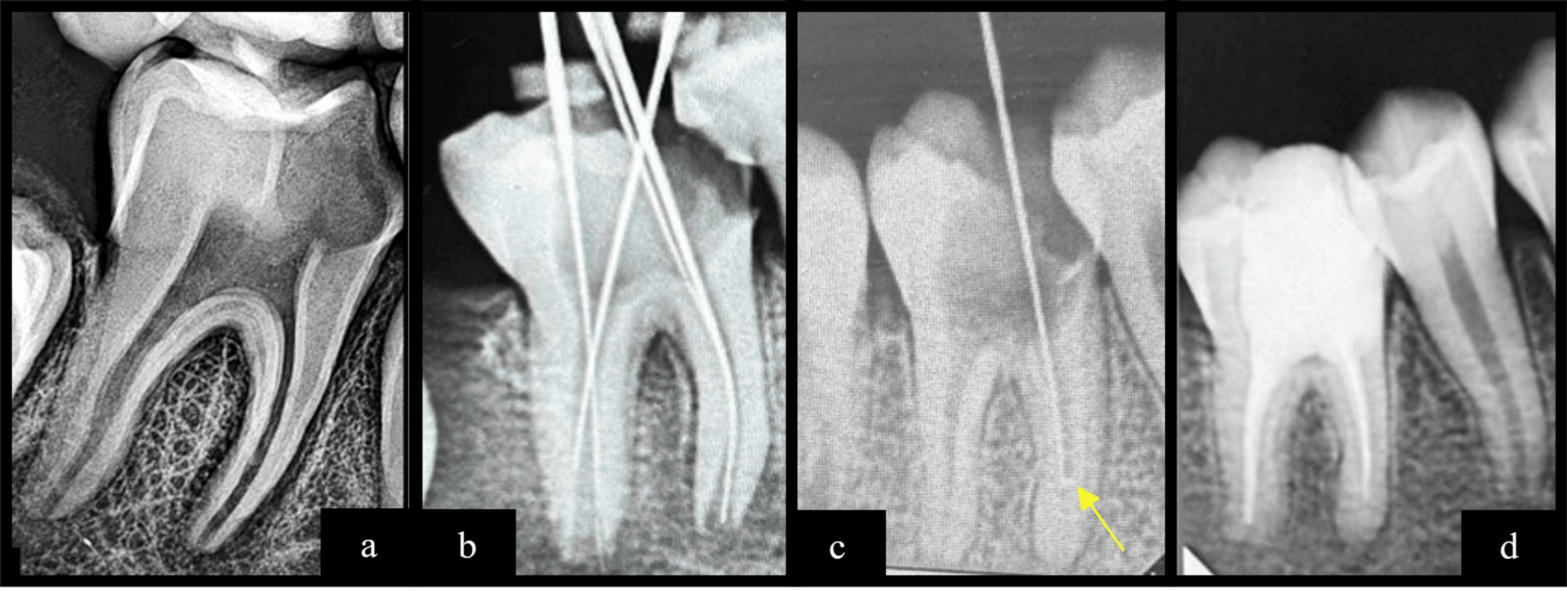
(a) Preoperative radiograph with respect to tooth #46. (b) Working length determination showing open apex. (c) Calcified distal canals after long-term Metapex intracanal dressing. (d) Completion of root canal therapy.

An eight-year-old male patient presented with a chief complaint of pain in the right lower back tooth region for one month. Clinical examination revealed a deep carious lesion in relation to tooth #46. Radiographic evaluation demonstrated an immature root apex with a periapical radiolucency. The tooth did not respond to electric or cold pulp testing, for which apexification was planned. At the first appointment, access cavity preparation and thorough cleaning of the root canals using normal saline irrigation were performed, and Metapex was placed as a long-term intracanal medicament, and the cavity was sealed with an interim restorative material. The patient failed to attend subsequent follow-up visits and reported after 10 months. At recall, the tooth was clinically asymptomatic, although the temporary restoration was dislodged. Radiographic evaluation revealed healing of the periapical lesion with bone formation and closure of the open apices. During re-instrumentation and canal debridement, a calcific barrier was observed in the distal root approximately 5-6 mm short of the apex, impeding further canal negotiation (Figure 2).

Case 3

**Figure 3:**
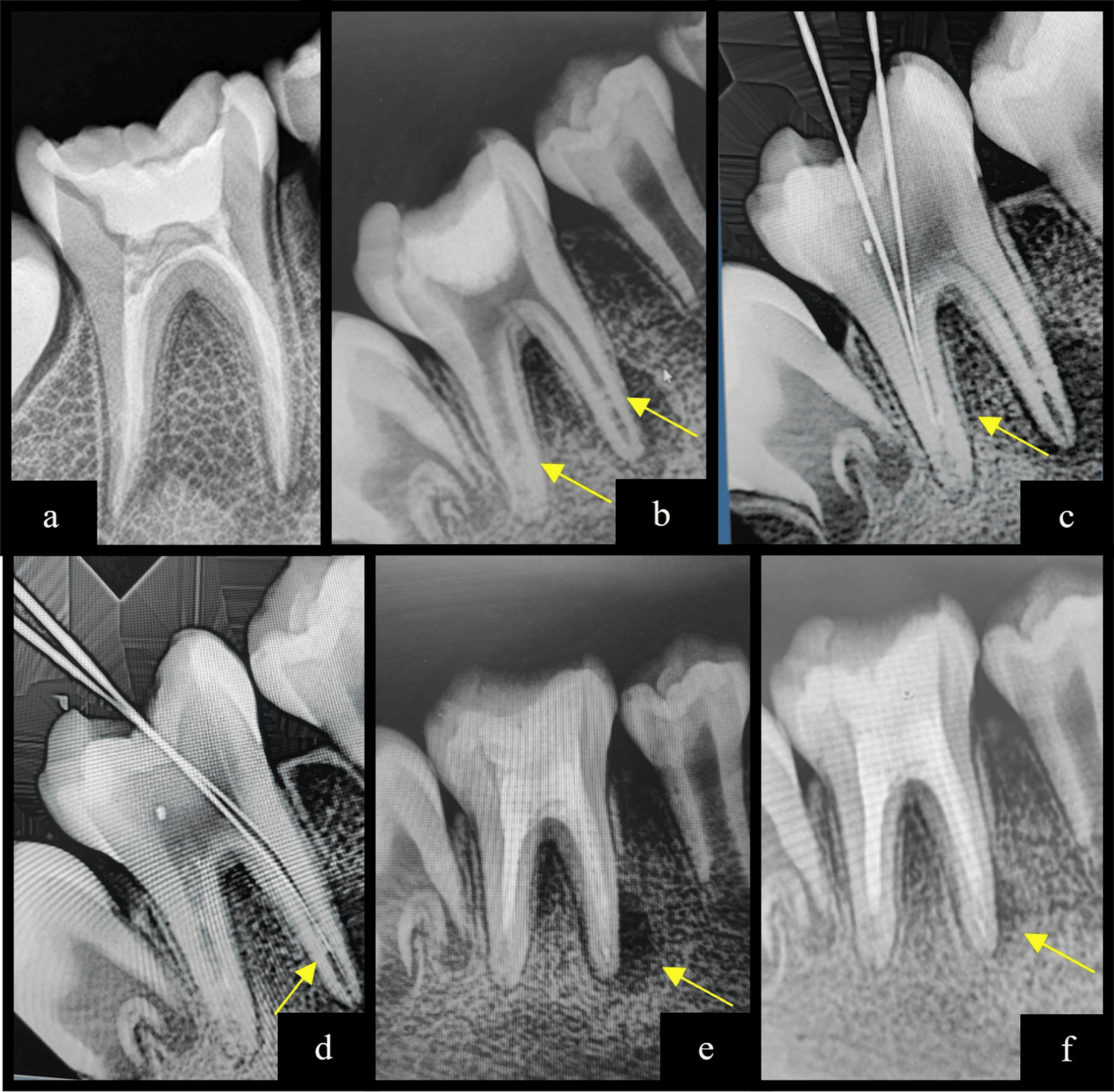
(a) Metapex dressing with respect to tooth #46. (b) Metapex removed with respect to tooth #46. (c, d) Working length determination showing calcification after Metapex removal. (e) Completion of root canal therapy. (f) Three-month follow-up showing evident healing of the lesion.

A nine-year-old male patient presented with a chief complaint of pain in the right lower molar region. The patient had a history of incomplete endodontic treatment of the mandibular right first molar (#46) approximately one year prior. On clinical examination, tooth #46 was found to be grossly carious, and RVG revealed partially treated root canals with residual radiopaque intracanal medicament, open apices, and a periapical radiolucency. The tooth was re-accessed under isolation, and thorough cleaning was performed using normal saline. During the irrigation procedure, remnants of yellow-colored material consistent with Metapex were observed extruding from the canals. After complete removal of the intracanal medicament, canal instrumentation revealed widespread hard tissue deposition in the apical third, which hindered further negotiation to the apex and interfered with working length determination (Figure 3).

Management

Under local anesthesia, a proper access opening was achieved using an Endo-Z bur (Dentsply Maillefer, Ballaigues, Switzerland). Canal patency was initially negotiated with a size #8 C+ file (Dentsply Maillefer), maintaining the instrument parallel to the long axis of the tooth at all times. Upon encountering the calcific barrier, the position of the file within the canal was confirmed radiographically. After verifying the correct position, the working length was established with the aid of another radiograph. A copious amount of 17% EDTA solution (Meta EDTA, Meta Biomed Co. Ltd.) was utilized to demineralize the calcified tissues and facilitate canal negotiation. A glide path was prepared using a size #10 C+ file (Dentsply Maillefer), following which cleaning and shaping of the canals were performed using hand instruments up to ProTaper size F1 (Dentsply Maillefer). During instrumentation, intermittent irrigation with normal saline was carried out to remove debris and maintain canal cleanliness. Following biomechanical preparation, calcium hydroxide (Waldent Calplus, Waldent Innovations Pvt. Ltd., New Delhi, India) was placed as an intracanal medicament, and the access cavity was temporarily sealed. At the two-week follow-up, the patient was asymptomatic, and obturation was performed using ProTaper gutta-percha cones (Meta Biomed Co. Ltd.) and zinc oxide eugenol sealer (Prevest DenPro Limited, Jammu, India). Finally, the tooth was restored with a high-strength posterior glass ionomer cement (3M ESPE Ketac Molar, 3M ESPE, St. Paul, MN, USA) as an interim post-endodontic restoration. At follow-up, all treated teeth were asymptomatic, nonmobile, and nontender on percussion, with no evidence of periodontal pathology. Radiographic evaluation demonstrated satisfactory healing of the periapical lesions.

## Discussion

When immature permanent teeth are affected by pulpal necrosis and chronic periapical periodontitis during root development, root maturation may be interrupted, resulting in short roots with thin, fragile dentinal walls. In such cases, there is an increased risk of root filling materials being extruded into the periradicular tissues, along with a heightened likelihood of root fracture [11]. The management of such cases advocates the use of two methods: either long-term apexification using calcium hydroxide and its combination with iodoform or apexification with the placement of an artificial apical barrier using MTA and Biodentine [12]. Ca(OH)₂ possesses a high alkaline pH, strong antimicrobial activity, and the ability to promote hard tissue formation, making it the gold standard for apexification despite requiring multiple visits and having the potential to weaken dentin with prolonged use [5]. The calcium hydroxide-iodoform combination, consisting of calcium hydroxide, iodoform, and silicone oil, acts as an antimicrobial, radiopaque, and resorbable paste commonly used in pulpectomy and apexification procedures [7]. MTA, a calcium silicate-based cement, is biocompatible, provides excellent sealing ability, and induces apical barrier formation, allowing single-visit apexification, although its high cost remains a limitation [1,2]. Similarly, Biodentine, composed of tricalcium silicate, calcium carbonate, and zirconium oxide, is a bioactive and biocompatible material with excellent sealing ability and a short setting time, making it an emerging material for apexification with favorable outcomes, especially in children [1,2].

Among these, the calcium hydroxide-iodoform combination has been widely adopted owing to its antimicrobial efficacy, radiopacity, ease of application and removal, and rapid resorption if extruded beyond the apex [5]. This combination forms the basis of commercially available, ready-to-use pastes such as Metapex, Vitapex, CalSeal Plus, Calplus, and RC Pex, which are extensively employed in pediatric endodontics [1].

The calcium hydroxide-iodoform combination is primarily indicated as a root canal filling material in primary teeth and as an intracanal medicament in permanent teeth for disinfection and healing of teeth with periapical infections. However, its use is contraindicated in patients with known allergy or hypersensitivity to any of its components [5,7].

There have been a number of reports describing the use of calcium hydroxide and iodoform paste (Metapex) in apexification. It has been successful in promoting root-end growth and apical closure in immature permanent teeth in children [13]. Lu and Qin (2004) and Weng (2004) reported the use of calcium hydroxide and iodoform paste (Vitapex) in apexification, showing the same level of radiographic success in young permanent teeth [14,15].

In the present case series, long-term apexification using calcium hydroxide and iodoform paste (Metapex) was carried out. Calcium hydroxide is capable of promoting the formation of a calcified barrier and hard tissue during procedures such as indirect and direct pulp capping, as well as apexification [5]. Its alkaline environment stimulates the activity of alkaline phosphatases, which release phosphate ions. These ions combine with calcium ions from the bloodstream to generate calcium phosphate, a key component in the development of calcified tissues [16].

All three cases demonstrated clinical and radiographic evidence of successful treatment outcomes, as evaluated by resolution of apical periodontitis, closure of the root apex, and healing of the surrounding bone. Unlike barrier formation, continued root growth was observed in all treated teeth. Similar findings were reported by Sridhar and Tandon (2013) [13] for Metapex and Gu et al. (2007) [17] for Vitapex paste, in which complete root development and apical closure were observed.

All three cases treated with apexification using calcium hydroxide with iodoform (Metapex) demonstrated intracanal calcification during long-term follow-up. The intracanal calcifications observed in the present cases were distinct from the intended apical calcific barrier formed during apexification, as these calcified deposits developed within the canal lumen and interfered with canal negotiation. Similar intracanal calcifications were reported in cases of REPs using calcium hydroxide as an intracanal medicament [16]. Song et al. (2017) [18] reported the incidence of intracanal calcification in revascularization and identified calcium hydroxide as a potential contributing factor.

The exact mechanism underlying intracanal calcification following apexification remains incompletely understood. Previous studies in regenerative endodontics have suggested that calcium hydroxide may influence hard tissue formation and cellular differentiation within the canal space [8,18]. In immature teeth with open apices, the periapical tissues may serve as a source of mesenchymal progenitor cells derived from the periodontal ligament and alveolar bone marrow, which possess the potential for cementogenic and osteogenic differentiation [19]. The migration and differentiation of these cells, combined with the bioinductive effects of calcium hydroxide, may contribute to ectopic calcified tissue deposition within the canal lumen [19,20].

The in vitro study by Jiang et al. (2023) [8] demonstrated that calcium hydroxide enhances both the migration and differentiation of dental pulp stem cells. Additionally, calcium ions stimulate the calcification of these cells. A similar association between complete calcification and Ca(OH)₂ pretreatment was reported by Almutairi et al. (2022) in their meta-analysis, in which 46.5% of cases of regenerative endodontic treatment demonstrated this finding [20].

The average span of missed appointments in the present cases was nine months. Jiang et al. (2023) [8] and Song et al. (2017) [18] proposed that the extent of root canal calcification may be linked to the length of the follow-up period. In the present case series, teeth treated with apexification using calcium hydroxide with iodoform (Metapex) showed root canal calcification during long-term follow-up. While this does not influence the long-term prognosis of the tooth, it can complicate future endodontic treatment required in apexification cases. A limitation of the present case series was the absence of standardized long-term follow-up intervals for all patients because of irregular patient compliance.

## Conclusions

This case series highlights intracanal calcification as a possible clinical finding associated with prolonged placement of calcium hydroxide-iodoform intracanal medicament in immature permanent teeth undergoing apexification. In the present cases, delayed follow-up and extended intracanal medicament duration were observed alongside partial canal obliteration and calcific changes that complicated subsequent canal negotiation.

Although a direct causal relationship cannot be established from this limited case series, the findings emphasize the importance of regular follow-up and careful monitoring during long-term apexification procedures. Further longitudinal and histopathological studies are required to better understand the pathogenesis and clinical implications of intracanal calcification following apexification.
